# Acting on better data for general medical care will help solve our acute hospital access crisis

**DOI:** 10.5694/mja2.51385

**Published:** 2022-01-24

**Authors:** Harvey H Newnham

**Affiliations:** ^1^ Alfred Health Melbourne VIC; ^2^ Monash University Melbourne VIC

**Keywords:** Health services research, Quality assurance, health care, Home care services, hospital‐based, Hospitals, Quality of health care

Smarter measures for general medicine are needed to improve hospital access

*“What gets measured gets done” — WE Deming*

Our acute hospitals should look to general medical units for help in solving our current hospital access crisis. They are where to find many beds that we so desperately need. General medical units are the largest users of acute, multiday inpatient beds, caring for up to one‐third of all adult multiday medical admissions in some states.^1^ A general medicine service in a metropolitan hospital typically has about 100 inpatients with an average length of stay of 5 or more days. Hence, as little as a 10%, or half a day, reduction in length of stay would provide ten beds for other patients without requiring infrastructure costs. Why is there so much talk about saving hours and minutes in emergency departments when general medicine lengths of stay are measured in days; an order‐of‐magnitude greater opportunity to release beds?

Such improvements should be achievable. Eleven percent of multiday inpatients in a Melbourne public hospital were recently categorised as no longer needing to be in an acute care environment, with many having the characteristics of general medicine patients.^2^ An additional proportion of general medicine patients could have received some of the acute phase of their care at home if better home‐based services had been in place.^2^ The paucity of this kind of published data regarding general medicine’s inpatient bed utilisation exemplifies how this specialty “flies under the radar” despite its central role in Australian health care. So why aren’t general medicine units at the front of the queue for support to help resolve our current access crisis?

One likely reason is the difficulty in providing meaningful data for evaluation of general medicine care.^3^ Compared with other large units such as intensive care, trauma services and many of the surgical specialties, general medicine lacks registry data to evaluate care and guide improvement.^4^ General medicine instead relies mainly on the usual suspects: hospital length of stay, readmission rates, inpatient mortality, individual ad hoc clinical audits, some data on hospital‐acquired complications, and compliance with our excellent national quality standards to evaluate its performance. Disease‐specific indicators for common chronic conditions such as heart failure and chronic obstructive pulmonary disease, although helpful, do not clearly reflect the impact of the multiple comorbidities including cognitive, social, addiction and mental health problems that are so common for general medicine patients.^3^

What data could help? The Box outlines a suggested framework of goals, actions and measures for improvement of general medicine care and bed utilisation. The framework is derived from the literature and my many conversations with local, national and international colleagues, guided by the principle that one should “Set up what you do to reinforce what it is you want to achieve” (personal communication, Matt Hill, United Kingdom National Health Service, June 2021).

Box 1 **General medicine actions and evaluations to add to standard practice**

**Table:** 

Goal	Specific actions	Evaluations
A. Develop a culture of excellence in meeting the needs of patients that increases the proportion of acute and chronic care that can safely be provided at home	▪ Create space for conversations that matter with inpatient and community staff (including GPs) and consumers to improve care ▪ Develop needs‐based patient assessment processes and tools that prompt timely involvement of clinicians in the patient journey before and after admission ▪ Increase inpatient clinician experience of home care ▪ Biennial feedback forums for consumers and carers with “lived experience” ▪ Ensure complex discharge planning and early intensive allied health therapy both happen in parallel with acute medical recovery, agnostic to the place of care ▪ Involve staff with expertise in home care when making acute care plans^2^ ▪ Develop a step‐up and step‐down acute general medicine team for virtual or face‐to‐face support to keep patients in or draw patients to home care, to provide real time support for, and share clinical risk with, GPs^5^	▪ Audit documentation of patients’ needs assessments ▪ Proportion of staff completing home visits in previous 2 years ▪ Evidence of consumer engagement and co‐design ▪ Consumer feedback relevant at unit level ▪ Hours of delay to first allied health therapy ▪ Functional outcomes^6^ ▪ Proportion of patients with primary care communication before presentation ▪ Time from readiness to actual discharge ▪ Necessity of admissions and ongoing days of stay* ▪ Appropriateness of care^1^, ^7^ ▪ Patient‐reported outcome measures for acute episodes of care* ^,^ ^8^
B. Make it easier for clinicians and patients to “do the right thing”	▪ Ensure adequate resourcing, infrastructure, level of experience and rostering for all general medicine clinical disciplines, inpatient and community, including after hours ▪ Simplify processes for referral and triaging to home‐based services ▪ Improve digital home monitoring ▪ Improve in‐reach to residential aged care facilities ▪ Develop methodology to identify, document and escalate delays to progression of care for inpatient and community patients ▪ Develop a bedside learning coordinator role focused on clinician improvement^9^ ▪ Develop relevant data dashboards for clinical staff ▪ Ensure geographic co‐location of general medicine patients with their teams ▪ Consider workload impacts when reviewing clinical incidents ▪ Optimise the electronic medical record — identify super‐user mentors to coach others	▪ Delay of acceptance and rejection rate by National Disability Insurance Scheme, and subacute and other admission substitution and ambulatory care services ▪ Delay to completion of subspecialty referral, imaging and procedures ▪ Identification of other critical bottlenecks and delays to progression of care ▪ Proportion of outlier patients ▪ Validated measurement of interdisciplinary teamwork and other non‐technical skills^10^ ▪ Validated assessment of staff wellbeing ▪ Rostered and unrostered overtime and personal leave
C. Encourage investment in general medicine by boards and health departments through better quality assurance	▪ Develop, with clinicians, agreed criteria for structured annual unit self‐assessment reports including risk assessments^11^ ▪ Periodically require independent review of safety, quality, effectiveness and value of care in general medicine units^12^	▪ Internally published general medicine unit self‐assessments ▪ Periodic internal audit of clinical services (consider Victorian Managed Insurance Authority^11^ or Dutch models^14^)

GP = general practitioner.

* Potential key performance measures.

The first goal is to develop a culture of excellence in meeting the acute and chronic needs of patients that also prioritises home‐first care. Many inpatient clinicians have never provided a home visit and are not fully conversant with the risks and benefits of home‐based care. To the uninitiated, general medicine care demands familiarity with a complex web of services, all with different acronyms and criteria for accepting patients. Inexperienced staff facing this complexity are at risk of procrastination or worse; making inappropriate referrals that cause long and costly delays in care. These staff are likely to make sounder decisions regarding home care if they have colleagues experienced in home care at their side.^2^ Specific actions and measures are proposed in the Box, with the most important measure being local development of a robust, independent estimate of the proportion of patients whose admission and ongoing days of stay might have been avoided.^2^ A similar approach should identify critical bottlenecks and barriers to progression of care, an essential component of improving flow.

The second goal is to make it easier for general medicine clinicians and patients to “do the right thing”. General medical units are rightly regarded as excellent in‐ and out‐of‐hours training rotations for medical interns, junior registrars and trainees of other disciplines. Junior staff need practicable jobs with sustainable rosters and workloads as well as good supervision. They should be counterbalanced by well resourced, experienced staff who have time to teach. A general medicine team to support acute step‐up and step‐down care in conjunction with home‐based services and general practitioners is needed.^5^ A bedside learning coordinator role should be explored to systematically gather experience‐based insights for improvement from frontline staff and rapidly foster cross‐team collaboration.^9^ Excellence in interdisciplinary teamwork and communication are core relational competencies for general medicine clinicians; we should adopt models that develop these competencies, and utilise measurement scales to evaluate them.^10^

The third goal is to encourage investment by health service boards and our Departments of Health in the resourcing and improvement of general medicine care to fast track these actions. As long as patients are not being harmed and staff are not breaking down, general medicine units do not get the attention that their activity warrants. Boards need to ask questions like: “How do we know how good our care is for complex medical patients?”, “How good are we at deciding who can receive care at home?” and “What systems and processes are in place to ensure that we are using our beds wisely?”.

Our general medicine units and their patients are integral components of the complex, adaptive systems of health care that require deep understanding for sustainable improvement. If health service boards and our state health departments choose to further extend their remit beyond “ensuring that ‘as few things as possible go wrong’ to ensuring that ‘as many things as possible go right’”,^13^ then using incisive measurement tools to uncover and act on opportunities to improve general medicine units (Box) is a good place to start. They will discover an abundance of discretionary energy to unlock the beds we need, to the benefit of our patients and dedicated staff.

During the recent crisis of coronavirus disease 2019 outbreaks in residential aged care facilities, the Victorian and Commonwealth Departments of Health rapidly collaborated with health services and many clinical disciplines to establish the Victorian Aged Care Response Centre.^15^ We need similar timely leadership, collaboration and investment in our general medicine units to help resolve this access crisis.

## Competing interests

No relevant disclosures.

## Provenance

Not commissioned; externally peer reviewed.
